# The Effects of a Fermented Rapeseed or/and Soybean Meal Additive on Antioxidant Parameters in the Blood and Tissues of Piglets

**DOI:** 10.3390/ani11061646

**Published:** 2021-06-01

**Authors:** Anna Czech, Iwona Sembratowicz, Martyna Kiesz

**Affiliations:** Department of Biochemistry and Toxicology, Faculty of Animal Sciences and Bioeconomy, University of Life Sciences in Lublin, Akademicka 13, 20-950 Lublin, Poland; anna.czech@up.lublin.pl (A.C.); martynatul@gmail.com (M.K.)

**Keywords:** fermented feeds, piglets, blood, tissues, redox status

## Abstract

**Simple Summary:**

The use of fermented products, including fermented soybean meal, and recently also fermented rapeseed meal, in the diet of pigs and other animals is becoming increasingly popular. A diet containing protein components from which anti-nutrients and allergenic substances have been eliminated by means of fermentation stimulates beneficial gastrointestinal microflora, resulting in improvement in metabolic processes and thus improved animal health.

**Abstract:**

The aim of the study was to assess the effect of fermented soybean meal (FSBM) and/or rapeseed meal (FRSM) on the redox status of blood and tissues in piglets. The experiment was conducted on 150 28-day-old weaned piglets divided into five groups. Piglets in the control group received standard diets with soybean meal. Animals in the experimental groups received diets in which a portion of the soybean meal was replaced with FRSM and/or FSBM: group FR—8% FRSM; group FR/FS—6% FRSM and 2% FSBM; group FS/FR—2% FRSM and 6% FSBM; and group FS—8% FSBM. Group FR/FS showed an increase in total antioxidant potential of plasma (FRAP) and low-molecular-weight antioxidants, i.e., vitamin C, urea, uric acid, and albumin, as well as an increase in catalase activity. Blood levels of lipid hydroperoxides (LOOH) and malonyl dialdehyde (MDA) were decreased. A reduction in lipid peroxidation due to the use of FR/FS was also indicated by a decrease in liver MDA and jejunum wall LOOH levels. Increases in superoxide dismutase (SOD) and catalase (CAT) activity and vitamin C levels in these tissues were also noted. The results of the study indicate that the inclusion of fermented rapeseed meal in the diet (6%) in combination with soybean meal (2%), improved the redox status of the weaners.

## 1. Introduction

One of the most commonly used protein components in feed for pigs is soybean meal (SBM). In the search for alternative sources of protein for animals, much attention has been focused on the possibility of using rapeseed meal (RSM) [1]. Rapeseed meal is a rich source of sulphur amino acids and minerals [2]. However, due to lower protein digestibility and lower lysine content compared to SBM, and presence of anti-nutritional factors (ANFs) such as tannins, phytic acid, crude fiber, and especially glucosinolates, the use of rapeseed meal in the diet of swine is limited [3]. Glucosinolate hydrolysis products have a goitrogenic effect, as they inhibit the synthesis of thyroid hormones and lead to thyroid gland hypertrophy [4]. The undesirable effects observed with these compounds may include suppression of feed intake and growth, especially in younger animals [5]. Soybeans also contain substances with anti-nutritional properties, such as galactooligosaccharides, tannins, phytates, lectins, trypsin inhibitors [6] and allergenic proteins (glycinin and β-conglycinin) [7]. Microbial fermentation is one effective method of eliminating ANFs from seeds [8]. The inclusion of fermented products may enrich the diet with beneficial microorganisms and thus also with the substances they produce, such as vitamins, enzymes, and organic acids [9]. Research by Yuan et al. [10] in piglets showed that fermented soybean meal (FSBM) had a beneficial impact on their growth performance, nutrient digestibility, and the fecal microbiome. A positive influence of the inclusion of FSBM in the diet of finishing pigs was reported by Feng et al. [11]. FSBM improved average daily gains, positively affected fecal microbial composition and meat quality. An earlier experiment conducted by Feng et al. [12] demonstrated that the addition of FSBM to the diet of weaned piglets increased activities of intestinal enzymes, improved nutrient digestibility and had beneficial influence on growth performance parameters. The introduction of FSBM in the diet of chickens resulted in an increase in the glutathione level and in the total antioxidant potential of the blood plasma [13]. Positive effects of the use of fermented rapeseed meal (FRSM) in sows and their offspring were reported by Grela et al. [14]. The inclusion of 4–9% FRSM in the diet of pregnant sows improved their gut microbiome, elevated immunoglobulins content in colostrum and increased production parameters, mainly in primiparous gilts, contributing to an increase in litter size and in litter weight at 28 day of age. A recent study by Satessa et al. [15] showed that the use of FRSM in the diet of weaned piglets may be a good alternative to zinc oxide in the prevention of post-weaning intestinal disorders. In a study in turkeys, the inclusion of fermented rapeseed cake in the diet in place of soybean meal [16] had a positive effect on the redox status of the breast muscles. One of the effects of fermentation is an increase in the amount of low-molecular-weight peptides that exhibit antioxidant properties [17]. Soybean fermentation was found to influence other valuable antioxidants, i.e., flavonoids, which are often present in the seeds in the form of glycosides. Fermentation may result in the release of aglycones, which not only have better antioxidant activity, but are also more digestible than the glucoside forms [17,18].

Therefore, there are indications that the inclusion of fermented components in place of SBM in the diet may positively affect the redox status of piglets and minimize oxidative stress. Hence, the aim of the study was to assess the effect of the addition of fermented components based on soybean and/or rapeseed meal on redox parameters in the blood and tissues of weaned piglets.

## 2. Materials and Methods

### 2.1. Animals and Experimental Design

The study was conducted on 150 28-day-old weaned piglets divided into 5 analogous groups in terms of body weight and sex. Each group comprised 30 weaned piglets (15 gilts and 15 barrows), which were placed in 15 pens with 2 piglets in each (1 gilt and 1 barrow). The piglets were housed in a building with controlled environmental conditions and received crumble feed in an identical feeding system to approximately 30 kg body weight. From 29 to 42 days of life the piglets were fed prestarter mixtures ad libitum. From day 43, they were fed starter diets ad libitum and kept in standard rearing conditions with constant access to fresh water.

Piglets in the control group (C) received standard complete prestarter and starter diets containing soybean meal (SBM). The experimental groups received feed in which a portion of the SBM was replaced with fermented dried rapeseed meal (FRSM) and/or fermented dried soybean meal (FSBM). Animals in group FR received feed with 8% FRSM; group FR/FS received a diet with 6% FRSM and 2% fermented FSBM; group FS/FR received a diet with 2% FRSM and 6% FSBM; and group FS received a diet with 8% FSBM. FRSM and FSBM were obtained from European Protein AS (Bække, Denmark). The piglets were fed dry compound feed in accordance with NRC [19]. The ingredient composition of the complete diets is presented in Table 1.

### 2.2. Laboratory Analyses

Dry matter, crude ash, crude protein, crude fat, and crude fiber were determined in the diets and in the fermented soybean and rapeseed meals [21]. Calcium content was determined by atomic absorption spectrometry with a Varian model 720-ES ICP-OES spectrophotometer (Varian, Palo Alto, CA, USA). Total *P* content in the feed and jejunum samples was determined colorimetrically with a Helios Alpha UV–Vis spectrophotometer (Spectronic Unicam, Leeds, UK). The contents of phytate phosphorus, lactic acid, glucosinolates, and tannins were determined in the diets [21].

Phytate was determined colorimetrically based on the pink colour of the Wade reagent, which is formed upon the reaction of ferric ions and sulfosalicylic acid and has an absorbance maximum at 500 nm. In the presence of phytate, iron is sequestered and is unavailable to react with sulfosalicylic acid, resulting in a decrease in the intensity of the pink colour [13,22].

The content of lactic acid was determined colorimetrically by the LA-Fe (III) complex method. A lactic acid–iron (III) complex was formed as a result of the acid–iron (III) chloride reaction, and its absorbance was measured at 410 nm using a Helios Alpha UV–Vis spectrophotometer (Spectronic Unicam, Leeds, UK) [23].

The glucosinolate content in the samples was estimated according to the ISO standard [24] by high performance liquid chromatography using the Agilent 1100 Series HPLC system, on an Agilent Zorbax ODS column (5 µL, 4.6 × 250 mm) with a UV–Vis detector (229 nm). The Folin–Denis spectrophotometric method was used to determine tannin content according to Canbaş et al. [25] with a modification.

At 77 days of age, blood from 5 piglets from each group was collected for analysis. One barrow was taken from each pen for blood sampling and slaughter (5 pigs/treatment). Piglets from all pens had similar body weight/pen. The animals were slaughtered in accordance with the technology currently employed in the meat industry with the use of electrical stunning, after which they were killed by bleeding by severing the blood vessels of the throat.

#### 2.2.1. Blood Analysis

The animals had no access to feed for 12 h before blood sampling. Blood was collected by a veterinarian from the jugular vein into heparinized 10 mL tubes. Biochemical parameters and mineral content were determined in the blood plasma, which was obtained by centrifuging whole blood at 3000 *g* for 10 min. Analyses were performed 3–4 h after the blood was collected. The blood was stored at 4 °C.

Test kits developed by Cormay (Lublin, Poland) were used to determine the content of total glucose (GLU), protein (TP), albumin (ALB), uric acid (UA), urea (UREA), creatinine (CREAT), and bilirubin (BIL). Ready-to-use test kits were used to determine the activity of selected enzymes: alkaline phosphatase (ALP), aspartate aminotransferase (AST), alanine aminotransferase (ALT), lactate dehydrogenase (LDH), and gamma-glutamyltransferase (GGT). The total antioxidant potential of the plasma (FRAP), level of malondialdehyde (MDA), lipid hydroperoxides (LOOH), and vitamin C, and activity of superoxide dismutase (SOD) and catalase (CAT) in the blood plasma were determined according to methods described by Sembratowicz et al. (13) and Czech et al. [26].

#### 2.2.2. Tissue Analysis

Tests were performed on the middle portion of the small intestine (jejunum), the liver, and the middle part of the longissimus thoracis muscle (LT). The levels of malondialdehyde (MDA), lipid hydroperoxides (LOOH), and vitamin C and the activity of superoxide dismutase (SOD) and catalase (CAT) in the tissues were determined according to methods described by Czech et al. [26].

### 2.3. Statistical Analysis

The numerical data were analysed by one-way analysis of variance (ANOVA) with orthogonal data, and the significance of differences between the groups was determined by the Tukey post hoc test, assuming significance levels of 0.05 and 0.01. The tables present the means and the standard error of the mean (SEM). The calculations were made using SAS 9.4 software (SAS Institute, Cary, NC, USA).

## 3. Results

The use of fermented components (FR, FR/FS, FS/FR, and FS) increased the TP content in the blood of piglets as compared to the control group (*p* ≤ 0.05). The highest TP concentration was recorded in group FR/FS. A significant increase in ALB content relative to groups C and FR was noted in the blood of piglets receiving a diet with FR/FS, FS/FR, or FS. The addition of FR/FS caused an increase in UA and UREA in the blood plasma in comparison to all other groups (*p* ≤ 0.05). A significant increase in GLU content relative to the control group was noted in group FS (Table 2).

Analysis of the effect of fermented components on the activity of selected enzymes revealed an increase in ALP and LDH in groups FR and FR/FS relative to the other experimental groups (*p* ≤ 0.05). The blood of piglets from the control group had significantly lower ALT activity than in groups FR and FR/FS and also of LDH and AST in comparison to all experimental groups (*p* ≤ 0.05) (Table 3).

The blood plasma of piglets receiving a diet with fermented rapeseed and soybean meal (group FR/FS) had significantly higher values of redox status parameters, i.e., CAT activity and FRAP, as compared to all other groups, as well as for vitamin C in comparison to the control and FR group (*p* ≤ 0.05). MDA and LOOH content in the control and group FS was significantly higher than in the other experimental groups (*p* ≤ 0.05) (Table 4).

SOD activity in the epithelium of the jejunum was significantly higher in groups FR, FR/FS and FS/FR than in the control (*p* ≤ 0.05). CAT activity was higher in groups FR and FR/FS than in groups FS/FR and FS and the control group (*p* ≤ 0.05). A similar pattern was observed in the longissimus thoracis muscle (LT). The content of vitamin C in the jejunum and LT muscle was significantly higher in the animals from groups FR and FR/FS, and additionally in the LT muscle of the animals from control group, compared to the other groups (*p* ≤ 0.05). The diet containing FR/FS and FS/FR significantly reduced the content of LOOH in the wall of the jejunum of piglets compared to the other groups (*p* ≤ 0.05). On the other hand, the concentration of LOOH in the LT muscle was significantly higher in the animals from group FS/FR vs FR/FS (*p* ≤ 0.05) (Table 5).

## 4. Discussion

Biochemical parameters of the blood reflect the state of the body and the changes taking place in it due to internal and external factors [27]. The total protein content in the plasma is one of the most important indicators of animal health. Urea is the main product of degradation of proteins of both exogenous (diet) and endogenous (muscles) proteins [28]. Changes in these parameters may reflect the course of metabolism of protein, as well as its utilization in animals. The inclusion of fermented components, especially fermented rapeseed meal with fermented soybean meal (FR/FS group), in the diets caused an increase in the total protein concentration in the blood, including that of albumins, as well as an increase in the amount of urea, the end product of their degradation. These results are probably the effect of the presence of fermented protein products of higher biological value, partially degraded proteins, and easily digestible essential amino acids [29]. The higher bioavailability of nutrients, mainly protein and essential amino acids, from fermented components has been demonstrated by Shi et al. [30].

The diet containing fermented meals also caused an increase in the activity of liver enzymes, ALT and AST in the blood of piglets. These enzymes are used as indicators, and an increase in their activity (especially ALT) in the blood can be a marker of liver damage [31]. On the other hand, it may indicate enhanced amino acids metabolism, especially given the increased levels of other blood parameters of, i.e., TP, ALB, and UREA. It should be noted, however, that the values of all biochemical parameters were within ranges considered to be physiologically normal [27]. The increase in activity of ALP, which is involved in ossification, in blood of pigs supplemented with fermented meals may have been due to increased intake of minerals taking part in this process, i.e., Ca and P [32]. Fermented products are known to be a rich source of enzymes of microbiological origin (e.g., phytase and non-starch polysaccharide hydrolysing enzymes), owing to which minerals are released from feed.

Analysis of the effect of the addition of fermented products to the diet of pigs on redox indicators of the blood revealed that the inclusion of fermented rapeseed meal with soybean meal in the diet (group FR/FS) had a beneficial effect on redox status parameters. In addition to an increase in catalase activity, there was also an increase in the concentrations of low-molecular-weight antioxidants, i.e., urea, uric acid, albumin, and vitamin C. According to Fang et al. [33], these are secondary endogenous antioxidants synthesized in numerous tissues (organs), thus constituting a protective barrier against the negative effects of free radicals. The increase in their concentrations in the blood raised the total antioxidant potential of the plasma (FRAP). This is a beneficial phenomenon, indicating better protection of cells and tissues against the negative effects of reactive oxygen species (ROS). A diet with fermented components, containing an increased number of short-chain peptides [17], may also contribute to an increase in the antioxidant capacity of the blood plasma. Both soybean and rapeseed are sources of other valuable antioxidants, i.e., polyphenols. Phenolic compounds present in rapeseed include phenolic acids, which may occur in a free form (mainly sinapic acid) or in form of choline ester of sinapic acid—sinapine [34]. In turn, the main representatives of polyphenols in soybeans are isoflavones (genistein and daidzein), which may occur in β-glycoside conjugates [35]. Fermentation not only can increase the content of polyphenolic substances (flavonoids and phenolic acids), but always may lead to elevation of compounds with higher antioxidant properties, i.e., flavonoid aglycones [17,18,36]. Thus, enrichment of the diet with these substances may also have increased the antioxidant potential of the plasma in the piglets from group FR/FS.

Owing to a well-functioning antioxidant system, the intensity of lipid peroxidation may have decreased in the weaners receiving FR/FS and FS/FR. This is indicated by the level of markers of lipid peroxidation in the plasma, i.e., LOOH and MDA. LOOH, as a by-product of this process, is closely linked to cell damage, and its level is generally regarded as an indicator of the degree of lipid peroxidation. MDA, on the other hand, is the end product of this process [37]. A study by Drażbo et al. [16] in turkeys showed that replacement of soybean meal with fermented rapeseed cake improved the antioxidant capacity of the breast muscles which was evidenced by a decrease in MDA contents and SOD and CAT activities.

The positive effects of the use of fermented components in the diet, mainly rapeseed meal (FR, FR/FS, and FS/FR), on antioxidant processes in piglets were also evident in the values of redox parameters determined in the cells of the jejunum wall and in the liver. The increase in SOD activity (in groups FR, FR/FS, and FS/FR) and CAT activity (in groups FR and FR/FS) in the cells of the jejunum wall and in the liver relative to the control group may have been linked to the presence of lactic acid bacteria [38]. According to Wang et al. [39] probiotic bacteria can be a source of low-molecular-weight antioxidants, including glutathione, butyrate, and folate. They can also contribute to an increase in SOD and GPx activities in serum as well as SOD activity in the muscles and CAT activity in the liver [40]. Due to the increase in enzymatic antioxidants and in the content of vitamin C (in the liver and jejunum) in the groups receiving FR in combination with FS, the level of markers of lipid peroxidation in these tissues was reduced. Replacement of soybean meal with fermented soybean meal (group FS) did not have such positive effects. The only beneficial effects were seen in the case of CAT activity and a reduced MDA level in the liver. Animals from group FS had significantly higher levels of LOOH (jejunum and liver) than animals from the other experimental groups, as well as lower SOD activity and vitamin C content than the animals whose diet contained fermented rapeseed meal. A study by Sembratowicz et al. [13] in chickens showed that FSBM only increased FRAP and glutathione level in the blood plasma but did not affect SOD or CAT activity or lipid peroxidation parameters. The reasons for better effect of FRSM supplementation on the values of redox indices in piglets in relation to FSBM may probably be due with the different content of antioxidant substances and their specific biological activity. So far, no comparative studies have been carried out in this regard, however, fermentation process may positively affect antioxidant activity of both meals.

## 5. Conclusions

The results of the study indicate that the inclusion of fermented rapeseed meal in the diet (6%) in combination with soybean meal (2%), improved the redox status of the weaners. This was manifested as an increase in FRAP and low-molecular-weight antioxidants, i.e., vitamin C, UREA, and ALB, as well as an increase in catalase activity. A decrease was noted in the level of markers of lipid peroxidation, LOOH and MDA, in the blood. A reduction in peroxidation of lipids due to the use of FR/FS was also indicated by a decrease in MDA in the liver and LOOH in the jejunum wall. In turn, an increase was noted in SOD and CAT activity and in the level of vitamin C in these tissues.

## Figures and Tables

**Table 1:** Ingredient composition (% of air-dry matter) of piglet diets and content of analysed nutrients and bioactive substances in 1 kg of FRSM (n = 4) and FSBM (n = 4) and piglet diets (n = 3).

Diet			Prestarter (29–42 Days)					Starter (43–77 Days)				
			Feeding Group *					Feeding Group *				
	FRSM	FSBM	C	FR	FR/FS	FS/FR	FS	C	FR	FR/FS	FS/FR	FS
Wheat			35.5	35.5	35.5	35.5	35.5	40.0	40.0	40.0	40.0	40.0
Barley			28.0	26.0	27.0	29.0	30.0	28.0	26.0	27.0	29.0	30.0
Soybean meal, 44% CP			16.0	10.0	9.0	7.0	6.0	16.0	10.0	9.0	7.0	6.0
Dried whey, 16% CP			4.0	4.0	4.0	4.0	4.0	0.0	0.0	0.0	0.0	0.0
Soybean oil			2.5	2.5	2.5	2.5	2.5	2.5	2.5	2.5	2.5	2.5
Complementary feed ^1–5^			8.5	8.5	8.5	8.5	8.5	8.0	8.0	8.0	8.0	8.0
Mineral–vitamin premix ^6^			5.0	5.0	5.0	5.0	5.0	5.0	5.0	5.0	5.0	5.0
Acidifier ^7^			0.5	0.5	0.5	0.5	0.5	0.5	0.5	0.5	0.5	0.5
FRSM			0.0	8.0	6.0	2.0	0.0	0.0	8.0	6.0	2.0	0.0
FSBM			0.0	0.0	2.0	6.0	8.0	0.0	0.0	2.0	6.0	8.0
Dry matter, g	882.7	901.1	891.4	889.5	890.2	889.9	889.8	890.2	888.9	889.4	889.5	889.1
Crude ash, g	78.9	66.1	50.68	50.67	50.66	50.69	50.72	50.42	50.51	50.41	50.56	50.57
Crude protein, g	291.8	499.7	187.8	187.0	187.5	188.0	188.1	181.0	181.1	181.1	181.1	181.1
Ether extract, g	31.7	35.6	50.31	50.32	50.34	50.35	40.33	50.13	50.18	50.14	50.15	40.13
Crude fiber, g	91.5	17.9	40.07	40.05	40.05	40.03	40.02	40.41	40.28	40.22	39.98	39.95
Metabolizable energy ^8^, MJ	12.27	15.64	13.32	13.32	13.31	13.32	13.32	13.21	13.21	13.21	13.21	13.21
Total phosphorus, g	9.09	7.12	6.81	6.82	6.82	6.81	6.81	6.78	6.75	6.78	6.76	6.77
Phytin phosphorus, g	5.73	4.61	3.92	2.44	2.73	2.82	2.74	3.93	2.46	2.76	2.87	2.79
Calcium, g	8.05	2.54	7.41	7.41	7.42	7.43	7.43	7.38	7.37	7.38	7.39	7.38
Glucosinolates, µmol g^−1^	6.37	0.92	0.081	0.330	0.174	0.103	0.062	0.081	0.331	0.175	0.104	0.063
Tannin, g kg^−1^	4.76	1.22	2.41	3.16	2.81	2.58	2.39	2.44	3.17	2.82	2.59	2.40
Lactic acid, g kg^−1^	50.42	44.22	14.21	81.54	80.16	75.83	74.94	14.01	81.34	79.93	75.15	74.65

FSBM—fermented dried soybean meal, FRSM—fermented dried rapeseed meal. * Feeding group, C—control; FR—group receiving a diet with 8% FRSM; FR/FS—group receiving a diet with 6% FRSM and 2% FSBM; FS/FR—group receiving a diet with 6% FSBM and 2% FRSM; FS—group receiving a diet with 8% FSBM. ^1^ Complementary feed, 1 kg (control group) containing crude protein 36.15%, lysine 2.50%, methionine 0.73%, crude fat 14.3%, crude fiber 2.85%, calcium 1.2%, phosphorus 0.85%, sodium 0.35%, BHT 280 mg; ^2^ Complementary feed, 1 kg (group FR) containing crude protein 36.65%, lysine 2.61%, methionine 0.75%, crude fat 14.6%, crude fiber 2.75%, calcium 1.15%, phosphorus 0.84%, sodium 0.33%, BHT 280 mg; ^3^ Complementary feed, 1 kg (group FR/FS) containing crude protein 36.60%, lysine 2.58%, methionine 0.76%, crude fat 14.5%, crude fiber 2.77%, calcium 1.17%, phosphorus 0.84%, sodium 0.33%, BHT 280 mg; ^4^ Complementary feed, 1 kg (group FS/FR) containing crude protein 36.25%, lysine 2.50%, methionine 0.75%, crude fat 14.3%, crude fiber 2.85%, calcium 1.2%, phosphorus 0.84%, sodium 0.35%, BHT 280 mg; ^5^ Complementary feed, 1 kg (group FS) containing crude protein 36.05%, lysine 2.49%, methionine 0.74%, crude fat 14.3%, crude fiber 2.90%, calcium 1.22%, phosphorus 0.85%, sodium 0.36%, BHT 280 mg; ^6^ 1 kg mineral–vitamin premix containing: calcium 130 g, phosphorus 50 g, sodium 35 g, magnesium 4.0 g, lysine 89 g, methionine 46 g, vitamin A 300,000 IU, vitamin D3 40,000 IU, vitamin E 2600 mg, calcium iodide 32 mg, selenium 8 mg, copper 3.2 g, iron 2.4 g, zinc 2.4 g, manganese 2976 mg, 25,000 FTU; ^7^ 1 kg acidifier containing orthophosphoric acid 320 g, citric acid 110 g, fumaric acid 50 g, propionic acid 45 g, formic acid 45 g, carrier (silicon dioxide) 430 g. ^8^ Metabolizable energy1 was calculated according to the equation proposed by Kirchgessner and Roth [20].

**Table 2:** Effect of fermented components on biochemical parameters of the blood plasma of piglets.

Parameter	Feeding Group *						
	C	FR	FR/FS	FS/FR	FS	SEM	*p*-Value
GLU; mmol L^−1^	4.75 ^b^	5.06 ^ab^	5.25 ^ab^	5.52 ^ab^	5.82 ^a^	0.112	0.033
TP; g L^−1^	51.75 ^c^	59.26 ^b^	67.73 ^a^	58.20 ^b^	58.94 ^b^	1.54	0.026
ALB; g L^−1^	39.71 ^b^	41.51 ^b^	46.48 ^a^	44.63 ^a^	45.35 ^a^	0.773	0.011
UA; µmol L^−1^	0.268 ^c^	0.291 ^c^	0.471 ^a^	0.403 ^b^	0.429 ^b^	0.023	0.034
UREA; mmol L^−1^	3.47 ^c^	4.92 ^b^	5.59 ^a^	4.06 ^c^	4.00 ^c^	0.177	0.046
CREAT; µmol L^−1^	109.9	101.9	108.6	112.6	100.6	1.81	0.121
BIL; µmol L^−1^	11.04	9.66	7.78	10.16	9.16	0.475	0.253

^a,b,c^—means with different superscripts within a row are significantly different at *p* ≤ 0.05; SEM—standard error of mean, * Feeding group abbreviations—see Table 1. Glu—glucose; TP—total protein; ALB—albumins; UA—uric acid; UREA—urea; CREAT—creatinine; BIL—bilirubin.

**Table 3:** Effect of fermented components on the activity of selected enzymes (U l^−1^) in the blood plasma of piglets.

Enzyme	Feeding Group *						
	C	FR	FR/FS	FS/FR	FS	SEM	*p*-Value
ALP	103.95 ^c^	173.20 ^a^	182.16 ^a^	161.40 ^b^	130.19 ^c^	5.83	0.009
ALT	25.80 ^b^	30.03 ^a^	30.12 ^a^	28.88 ^ab^	27.79 ^ab^	0.752	0.025
AST	26.39 ^b^	32.87 ^a^	34.08 ^a^	34.40 ^a^	32.79 ^a^	0.879	0.017
LDH	1311.4 ^c^	1539.9 ^a^	1592.6 ^a^	1437.1 ^b^	1475.7 ^b^	52.56	0.028
GGT	24.57	21.86	22.39	24.32	24.13	0.787	0.109

^a,b,c^—means with different superscripts within a row are significantly different at *p* ≤ 0.05; SEM—standard error of mean, * Feeding group abbreviations—see Table 1. ALP—alkaline phosphatase, ALT—alanine aminotransferase, AST—aspartate aminotransferase, LDH—lactate dehydrogenase; GGT—gamma-glutamyltransferase.

**Table 4:** Effect of fermented components on redox status parameters of the blood plasma of piglets.

Parameter	Feeding Group *						
	C	FR	FR/FS	FS/FR	FS	SEM	*p*-Value
SOD; U mL^−1^	78.38	78.85	79.14	79.52	79.24	0.129	0.247
CAT; U mL^−1^	3.69 ^b^	3.23 ^bc^	5.09 ^a^	3.86 ^b^	3.58 ^bc^	0.157	0.015
FRAP; µmol L^−1^	7.69 ^b^	6.70 ^b^	9.10 ^a^	6.43 ^b^	7.83 ^b^	0.222	0.022
Vitamin C; µmol L^−1^	21.48 ^b^	20.07 ^b^	27.21 ^a^	23.07 ^ab^	24.52 ^ab^	0.673	0.029
MDA; µmol L^−1^	6.93 ^a^	5.22 ^b^	5.68 ^b^	5.27 ^b^	6.44 ^a^	0.181	0.034
LOOH; µmol L^−1^	4.48 ^a^	3.88 ^ab^	3.36 ^b^	3.50 ^b^	4.33 ^a^	0.107	0.041

^a,b,c^—means with different superscripts within a row are significantly different at *p* ≤ 0.05; SEM—standard error of mean, * experimental group abbreviations—see Table 1. SOD—superoxide dismutase, CAT—catalase, FRAP—total antioxidant potential of plasma, MDA—malondialdehyde, LOOH—lipid hydroperoxides.

**Table 5:** Effect of fermented components on redox status parameters of selected tissues of piglets.

Parameter	Tissue	Feeding Group *						
		C	FR	FR/FS	FS/FR	FS	SEM	*p*-Value
SOD U g protein^−1^	jejunum	19.17 ^b^	21.08 ^a^	21.08 ^a^	20.82 ^a^	20.10 ^ab^	0.459	0.013
	liver	11.16 ^c^	13.37 ^a^	13.04 ^a^	13.23 ^a^	12.49 ^b^	0.347	0.036
	LT muscle	11.68	12.01	12.14	12.06	12.05	0.441	0.099
CAT U g protein^−1^	jejunum	4.83 ^b^	6.01 ^a^	6.78 ^a^	4.98 ^b^	5.38 ^b^	0.184	0.032
	liver	9.05 ^c^	28.85 ^a^	31.20 ^a^	23.48 ^b^	23.95 ^b^	1.51	0.019
	LT muscle	7.17 ^c^	10.70 ^a^	10.39 ^a^	8.79 ^b^	8.21 ^bc^	0.291	0.028
Vitamin C pmol mg^−1^	jejunum	190.5 ^c^	222.9 ^a^	215.6 ^a^	209.6 ^b^	197.7 ^c^	2.37	0.024
	liver	983.7 ^b^	1241.7 ^a^	1264.6 ^a^	1093.6 ^ab^	983.0 ^b^	5.62	0.037
	LT muscle	82.14 ^a^	84.86 ^a^	89.19 ^a^	31.95 ^c^	67.14 ^b^	4.49	0.008
LOOH µmol mg^−1^	jejunum	4.45 ^ab^	4.33 ^ab^	2.31 ^c^	4.24 ^b^	4.93 ^a^	0.180	<0.001
	liver	2.18 ^b^	2.07 ^b^	2.87 ^b^	2.47 ^b^	3.88 ^a^	0.147	0.023
	LT muscle	0.880 ^ab^	0.951 ^ab^	0.786 ^b^	1.092 ^a^	0.857 ^ab^	0.033	0.005
MDA nmol mg^−1^	jejunum	0.614	0.531	0.590	0.538	0.532	0.018	0.261
	liver	5.26 ^a^	2.48 ^c^	2.36 ^c^	2.57 ^c^	3.44 ^b^	0.209	0.022
	LT muscle	1.514	1.106	0.977	1.011	0.943	0.111	0.087

^a,b,c^—means with different superscripts within a row are significantly different at *p* ≤ 0.05; SEM—standard error of mean, * Feeding group abbreviations—see Table 1. SOD—superoxide dismutase, CAT—catalase, MDA—malondialdehyde, LOOH—lipid peroxides, LT muscle—longissimus thoracis muscle.

## Data Availability

All relevant data are included in the paper.
